# Age-dependent impairment of dopamine D1 receptor signalling in mouse striatum by *FMR1* variant P626L

**DOI:** 10.1093/braincomms/fcaf338

**Published:** 2025-09-10

**Authors:** Junyi Fu, Wei Jiang, Liping Shen, Jiaming Fu, Xianlai Duan, Detian Liu, Jingyi Long, Shunhua Ye, Lingjia Tang, Yong-Hong Yi, Yue-Sheng Long

**Affiliations:** Department of Neurology, Institute of Neuroscience, Key Laboratory of Neurogenetics and Channelopathies of Guangdong Province and Ministry of Education of China, The Second Affiliated Hospital of Guangzhou Medical University, Guangzhou, Guangdong 510260, China; Department of Neurology, Institute of Neuroscience, Key Laboratory of Neurogenetics and Channelopathies of Guangdong Province and Ministry of Education of China, The Second Affiliated Hospital of Guangzhou Medical University, Guangzhou, Guangdong 510260, China; Department of Neurology, The Third People’s Hospital of Huizhou, Affiliated Hospital of Guangzhou Medical University, Huizhou, Guangdong 516002, China; Department of Neurology, Institute of Neuroscience, Key Laboratory of Neurogenetics and Channelopathies of Guangdong Province and Ministry of Education of China, The Second Affiliated Hospital of Guangzhou Medical University, Guangzhou, Guangdong 510260, China; School of of Life Sciences and Biopharmaceutics, Guangdong Pharmaceutical University, Guangzhou, Guangdong 510006, China; Department of Neurology, Institute of Neuroscience, Key Laboratory of Neurogenetics and Channelopathies of Guangdong Province and Ministry of Education of China, The Second Affiliated Hospital of Guangzhou Medical University, Guangzhou, Guangdong 510260, China; Department of Neurology, The Third Hospital of Changsha, Hunan 410015, China; Department of Neurology, Institute of Neuroscience, Key Laboratory of Neurogenetics and Channelopathies of Guangdong Province and Ministry of Education of China, The Second Affiliated Hospital of Guangzhou Medical University, Guangzhou, Guangdong 510260, China; Department of Neurology, Longgang District Central Hospital of Shenzhen, Shenzhen, Guangdong 510120, China; Department of Neurology, Institute of Neuroscience, Key Laboratory of Neurogenetics and Channelopathies of Guangdong Province and Ministry of Education of China, The Second Affiliated Hospital of Guangzhou Medical University, Guangzhou, Guangdong 510260, China; Department of Human Genetics, Donders Institute for Brain, Cognition and Behavior, Radboud University Medical Center, Nijmegen 6525 GA, The Netherlands; Department of Neurology, Institute of Neuroscience, Key Laboratory of Neurogenetics and Channelopathies of Guangdong Province and Ministry of Education of China, The Second Affiliated Hospital of Guangzhou Medical University, Guangzhou, Guangdong 510260, China; Department of Emergency, Sun Yat-sen Memorial Hospital, Sun Yat-sen University, Guangzhou, Guangdong 518116, China; Department of Neurology, Institute of Neuroscience, Key Laboratory of Neurogenetics and Channelopathies of Guangdong Province and Ministry of Education of China, The Second Affiliated Hospital of Guangzhou Medical University, Guangzhou, Guangdong 510260, China; Department of Geriatrics, The Second Hospital of Ningbo, Ningbo, Zhejiang 315010, China; Department of Neurology, Institute of Neuroscience, Key Laboratory of Neurogenetics and Channelopathies of Guangdong Province and Ministry of Education of China, The Second Affiliated Hospital of Guangzhou Medical University, Guangzhou, Guangdong 510260, China; Department of Neurology, Institute of Neuroscience, Key Laboratory of Neurogenetics and Channelopathies of Guangdong Province and Ministry of Education of China, The Second Affiliated Hospital of Guangzhou Medical University, Guangzhou, Guangdong 510260, China

**Keywords:** *fragile X messenger ribonucleoprotein 1* gene, fragile X–associated tremor/ataxia syndrome, dopamine receptor, missense mutation, parkinsonism

## Abstract

Non-coding CGG repeat expansions in *fragile X messenger ribonucleoprotein 1* (*FMR1*) gene lead to fragile X–related disorders. Other than the CGG repeat expansion, several *FMR1* coding variants have recently been identified to impair the molecular functions of fragile X messenger ribonucleoprotein 1 (FMRP), implicating in FMR1-associated phenotypes. This study aims to investigate the pathogenic role of a novel *FMR1* missense variant from a parkinsonism patient without the typical CGG repeat expansion. Pathogenicity of the FMRP-P626L mutant was predicted using *in silico* analysis and structural prediction. A mouse model of FMRP-P608L mutation matched with the human FMRP-P626L was established. The effects on dopamine pathway in FMRP-P608L mice were investigated using behavioural test, immunohistochemistry, ELISA, quantitative PCR (qPCR), western blotting, co-immunoprecipitation and pharmacological intervention. We identified a missense variant (c.1877 C>T, p.P626L) in coding region of *FMR1* gene from a patient diagnosed with progressive rigidity and bradykinesia, which was predicted to be a damaging mutation. The corresponding mutation (P608L) mice at 6 months old exhibited impaired motor behaviours and decreased in striatal dopamine level in an age-dependent fashion. The mutant reduced FMRP binding to G protein–coupled receptor kinase 2 (GRK2), which resulted in abnormal localization of GRK2 and impairment of dopamine D1 receptor (D1R) pathways. Administration of D1R agonist rescued the motor disabilities observed in the mutation mice. This is the first report linking a point mutation in *FMR1* to parkinsonism, demonstrating that the FMRP-P608L mutation impairs the D1R pathway by reducing its binding to GRK2. Our findings enhance the understanding of pathogenic mechanisms underlying selective functional impairment by mutations.

## Teaser

Fu *et al*. report a novel *FMR1* missense variant (c.1877 C>T, p.P626L) from a patient diagnosed with parkinsonism. They demonstrate that this mutation impairs the dopamine D1 receptor (D1R) pathway by reducing its interaction with G protein–coupled receptor kinase 2, and treatment with D1R agonist alleviates the motor disabilities in mutation mice.

## Graphical Abstract

**Graphical Abstract:**
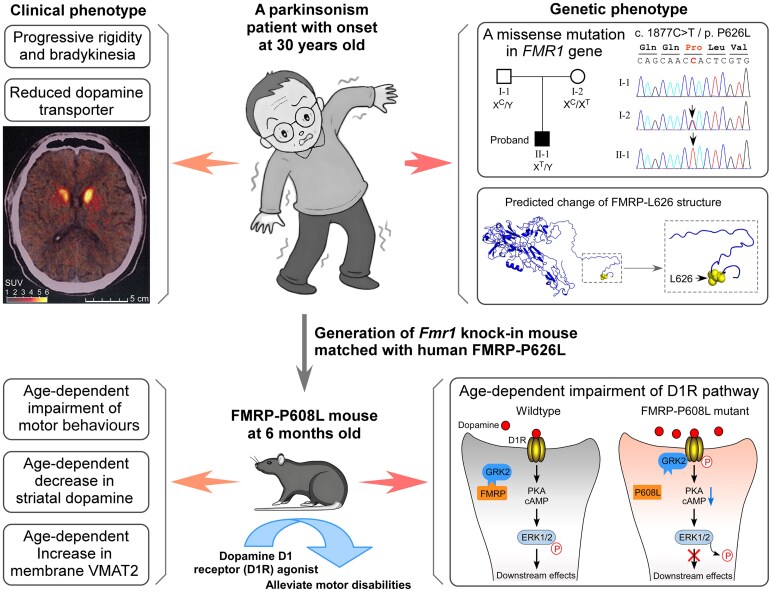

## Introduction

Fragile X mental retardation gene (*FMR1*), located at the Xq27.3 locus, is highly expressed in the nervous system and plays crucial roles in brain development, neuronal differentiation, and synaptic plasticity.^1-3^ A full mutation with over 200 CGG trinucleotide repeats in the 5ʹ untranslated region (5ʹ UTR) of this gene results in a common neurodevelopmental disorder known as fragile X syndrome (FXS)^4,5^; and a pre-mutation within a range from 55 to 200 CGG repeats leads to Fragile X–associated tremor/ataxia syndrome (FXTAS), a progressive neurodegenerative disorder characterized by adult-onset tremor, cerebellar gait ataxia, parkinsonism and neuropathy.^6-9^ Additionally, a high prevalence of parkinsonism phenotypes has also been identified from some grey-zone carriers with 45–54 CGG repeats in the *FMR1* gene, and some of them are manifested as a mimic of typical Parkinson’s disease.^10,11^ During recent years, an increasing number of rare variants including point mutations, deletions and duplications have been identified from some patients with or without intellectual disabilities,^12,13^ suggesting a wide and complex relationship between the *FMR1* variants and the diverse phenotypes of fragile X–related neurological disorders.

Fragile X messenger ribonucleoprotein 1 (FMRP) encoded by the *FMR1* gene acts as an RNA-binding protein to selectively transport mRNA from nucleus to synapse and negatively control local mRNA translation, which contribute to neural development and synaptic plasticity.^14-16^ The FMRP protein is involved in the regulation of gene expression, synaptic formation and functions and dopamine (DA) signals by interacting with other proteins (such as PABP1, RanBPM, GRK2 and CYFIP, etc.) with some functional domains at the N-terminal, nuclear localization signal (NLS), or C-terminal regions of this protein.^17-20^ Several rare variants within the FMRP functional domains have been identified to result in molecular dysfunctions and FXS-related phenotypes. For example, two mutations I304N and G266E located within the RNA-binding KH2 domain of the FMRP were identified to be loss of function linked to translational dysregulation of target mRNAs.^13^ R138Q at the RNA-binding KH0 domain of FMRP did not affect its RNA-binding function but selectively impaired the presynaptic functions through protein–protein interactions.^21^ Focusing on FMRP-interacting protein networks, a recent study revealed that most of the identified proteins tend to interact with the C-terminal region of the FMRP, suggesting an important role of the C-terminal region in the complex FMRP protein networks.^22^ Therefore, those C-terminal variants of the FMRP identified from patients should be worthy of investigation.

It is known that decreased DA level or impaired DA signalling pathways in striatum, a critical brain area harboured a population of dopaminergic neurons, has been demonstrated to contribute to motor dysfunctions in Parkinson’s disease.^23,24^ Restoring DA level and its downstream signalling could alleviate parkinsonian and dyskinetic states, and dopamine D1 receptor (D1R) represents a major target in the treatment of this disease.^25^ Accumulating evidences show that G protein–coupled receptor kinase 2 (GRK2) induces D1R phosphorylation in rat striatum,^26^ and the GRK2-mediated D1R phosphorylation further modulates a variety of downstream signalling pathways including protein kinase A (PKA) activation and increased cyclic adenosine monophosphate (cAMP) level linked to DA’s functions.^27^ A few studies have suggested that FMRP participates in the D1R signalling pathway by interacting with GRK2 in mouse forebrain, which are considered to contribute to some FXS-related phenotypes.^28,29^ However, the pathophysiological roles of *FMR1* mutations in parkinsonism are poorly known.

In this study, we screened a missense mutation (NM_002024.6: c.1877C>T, p.P626L) in the coding region of the *FMR1* gene from one male patient diagnosed with parkinsonism. Both clinical and radiological assessments of this patient indicated a reduced dopaminergic function. A previous study has determined that the FMRP plays crucial roles in DA modulation and DA-related behaviours by interacting with GRK2.^18^ Therefore, we hypothesize that the P626L mutation might affect the DA signalling pathway and motor behaviours though impairing FMRP’s binding to GRK2. By generating a mouse model of FMRP-P608L mutation matched with the human FMRP-P626L, this study aims to investigate the effects of this mutation on molecular and behavioural phenotypes, which will provide novel insights into the pathogenesis of fragile X–related parkinsonism.

## Materials and methods

### Patient

The patient, who complained of progressive rigidity and bradykinesia, was recruited from the Second Affiliated Hospital of Guangzhou Medical University. Detailed clinical information, including age of onset, clinical symptoms and medical therapy, was collected. MRI scanning was conducted to identify morphological abnormalities of the brain. PET/CT was conducted to evaluate the function and activity of the DA system by ^11^C-2-beta-carbomethoxy-3-beta-(4-fluorophenyl) tropane at a dose of 10.0 mCi.

The studies involving human participants were reviewed and approved by the institutional committee of the Second Affiliated Hospital of Guangzhou Medical University. The participants (the patient and his parents) provided their written informed consent to participate in this study.

### Whole exome sequencing and *in silico* analyses

Both the patient and his parents were examined by whole exome sequencing. The collection of genomic DNAs from blood samples of the participants was conducted for the purpose of segregation analysis. Subsequently, Whole Exome Sequencing (WES) was performed using the NextSeq 2000 (Illumina, San Diego, CA, USA). The sequencing data were obtained through the utilization of massive parallel sequencing, achieving an average depth exceeding 125 times and coverage of the capture regions surpassing 98%. Sequence alignment and variant calling were executed in accordance with established protocols as previously documented.^23^ The frequencies of the identified variants were retrieved from the Genome Aggregation Database, which includes data from East Asian and general populations.

Some Parkinson-related genes (Parkin/PARK2, PINK1, DYT1 and DJ1/PARK7) were examined due to the patient exhibited parkinsonism-like phenotype. The pathogenicity and allele frequency were predicted by several *in silico* tools including SIFT, Polyphen-2, Mutation Master, LRT, VEST3, M-CAP, CADD and DANN. Iterative Threading ASSEmbly Refinement (I-TASSER) was applied to predict the protein 3D structure (https://zhanglab.ccmb.med.umich.edu/I-TASSER/),^24-26^ and IUPred2A was used to predict protein–protein interaction.^27^

### Generation of FMRP-P608L mutation mice

*Fmr1* mutation mice were constructed using a congenic C57BL/6 strain. *Fmr1* missense mutation c.1823C>T mice (Pro608Leu) were generated by Cyagen Biosciences Inc. with CRISPR/Cas9 (Clustered regularly interspaced short palindromic repeats/ CRISPR-associated protein 9)-mediated genome engineering. This point variant is located on Exon 17 of the mouse *Fmr1* (NM_008031.3; Ensembl: ENSMUSG00000000838), and P608L mutation mice were constructed by in-house animal facility by coinjecting with Cas9 mRNA, gRNA and a donor oligo (gRNA1, 5′-CGTAGATGGGCTGCAACCGCTG G-3′, gRNA2, 5′-TAGGGTACTCCATTCACCAGCGG-3′ and donor oligo sequence, 5′-CGCACGGGTAAAGATCGTAACCAGAAGAAGGAAAAGCCAGACAGCGTAGATGGCTGCAACTGCTGGTGAATGGAGTACCCTAAATAAGCTACATAATTCCGAAGTTATATTTCTCTACCAT-3′) into fertilized eggs. After co-injection, the mice were genotyped by polymerase chain reaction (PCR), followed by sequence confirmation (*Fmr1*-F, 5′-CTAAACCATTACAGAGTGCCTCCAGT-3′; *Fmr1*-R, 5′-ATCCTGCCCTGAAG TGTTAAGTGTAC-3′). All mice were housed at a controlled temperature (25°C) and a 12-h light/12-h dark cycle, and only male mice were used for the following studies. All animal experiments had been approved by the Animal Ethics Committee of the Second Affiliated Hospital of Guangzhou Medical University.

### Behavioural analyses

(i) Rotarod test: the mice were tested for duration on the rotating bar at different rotation rates (19, 26 and 38 rpm/min). The maximum duration was limited to 5 min. Each mouse was recorded for three times at an interval of 1 min, and the average was calculated.^28^ (ii) Pole test: the pole included a thin wooden cylinder (length: 50 cm, diameter: 1.5 cm) and a cross-shaped wooden base. Rubber bands were wrapped around the cylinder to increase traction. The mice were put on top of the rod facing downwards, and the latencies of their dropping rod were measured. Each mouse was tested five times, and the lowest latency to descend the pole was analysed.^29^ (iii) Narrow beam test: this experiment consisted of a start-and-goal box crossed with a narrow beam (height: 100 cm, length: 100 cm and wide: 1 cm). Mice were trained to pass through the beam 10 times per day at an interval of 2 min. The latency was recorded according to the time from the start point to the terminal box.^30^ (iv) Swim test: the mice were placed in a container of water with 20 × 30 × 20 cm size at the temperature of 22–25°C. Each mouse was scored and recorded at 1 min intervals for 10 min.^31^ (v) Wire suspension test: the device consisted of a wire (length: 80 cm and height: 25 cm), which was fixed horizontally between two platforms. The paws of each mouse were hung in the middle of the wire, and the time of reaching one platform was recorded. The maximal time allowed was set at 120 s.^29^

Y-maze and step-down tests were selected for monitoring memory behaviours. Y-maze test was used for spontaneous alternation tasks. Animals were allowed 5 min to explore the maze during which their spontaneous alternations were recorded for later offline extraction using video. The measure of the animal working memory is the percentage of the possible complete triplets made during the set time [triplets/(total arm entries−2) × 100].^32^ The mice with long latency and few mistakes have strong ability of learning and memory in step-down test. In the training day, the mice were shocked (1 mA, 5 s) after they stepped down from an elevated platform. On the next day, the mice stayed on the same platform for a longer time before stepping down. Error times represent the number of times of the mice jumping off a platform.^33^ To more intuitively reflect phenotype, wild-type (WT) and FMRP-P608L groups each had 12 mice for behavioural tests.

### Tyrosine hydroxylase immunohistochemistry analysis

The WT and P626L mice were deeply anaesthetized using pentobarbital (120 mg/kg, i.p.) and subsequently perfused transcardially with 0.1 M phosphate-buffered saline (PBS, pH 7.4), followed by 4% paraformaldehyde in 0.1 M PBS. All brains were post-fixed by submersion in the same fixative for 24 h, after which they were cryoprotected in 10% sucrose for 24 h, followed by 30% sucrose for an additional 24 h. The brains were sectioned coronally at 50 μm using a sliding microtome. Immunohistochemistry was performed using the avidin–biotin complex peroxidase method. The sections were incubated overnight at 4°C with rabbit polyclonal anti-tyrosine hydroxylase (TH) (1:2000, Cat#: AB152, Millipore, RRID: AB_390204). The reaction product was visualized using a DAB (Diaminobenzidine) peroxidase substrate kit (SK-4100; Vector Laboratories, Burlingame, CA, USA). The TH-immunopositive cells in the ipsilateral and contralateral subfields of the corpus striatum and substantia nigra compact were quantified from three sections per animal. The number of immunoreactive cells was counted to determine their distribution density. Only cells with reaction products that exhibited a clear border were quantified from three non-overlapping fields under a light microscope at 200× magnification, with results presented as the average cell number per field for each section, using ImageJ software (NIH, Bethesda, MD, USA).

### Enzyme-linked immunosorbent assay

Enzyme-linked immunosorbent assay (ELISA) was used to measure mice tissues including cAMP, levodopa (L-dopa) and 3,4-dihydroxyphenylacetic acid (DOPAC). The shredded tissues were homogenized on ice with 1× PBS buffer. Homogenates were centrifuged at 4°C for 5 min, and the supernatants were assayed on an enzyme-labeled instrument (450 nm) using an ELISA kit to determine the levels of cAMP (CAT#: CEA003Ge, Cloud-Clone Corp.), L-dopa (CAT#: MBS9343615, MyBioSource) and DOPAC (CAT#: SL0850Mo, SUNLONG, China). Experimental procedures were performed according to the manufacturer’s instructions.

### Striatal dopamine measurement

The WT and P608L mice (*n* = 8) were euthanized by cervical dislocation, and the head was rapidly removed with scissors and then the striatum was rapidly dissected from each brain on ice. Dopamine concentration was determined in the striatal tissues using a mouse DA ELISA kit (Cusabio Biotech, Wuhan, China), following the manufacturer’s instructions. The absorbance of each well was measured at a wavelength of 450 nm using a 96-well microplate spectrophotometer (Multiskan GO, Thermo Scientific, Waltham, MA, USA).

### Membranous-cytoplasmic fractionation

Brain fractionation was performed using the Mem-PER^TM^ Plus Membrane Protein Extraction Kit (Thermo Scientific) following the manufacturer’s instructions. The striatum was rinsed and resuspended in Cell Wash Solution and then pelleted at 1000 × *g* before being resuspended in ice-cold permeabilization buffer and transferred to a new tube. After a 10-min incubation on ice, the sample was centrifuged at 16 000 × *g* for 15 min to pellet the permeabilized cells. The supernatant (cytosolic proteins) was carefully removed and transferred to a new tube. The insoluble (pellet) fractions were resuspended in an ice-cold solubilization buffer, and the mixture was pipetted up and down to achieve a homogeneous suspension, which was then incubated at 4°C for 30 min. The samples were subsequently centrifuged at ∼16 000 × *g* for 10 min, and the supernatant, representing the nuclear extract, was transferred to a new tube, containing solubilized membrane and membrane-associated proteins.

### Real-time quantitative reverse transcription polymerase chain reaction

Total RNA samples from mice brain tissues were isolated using a HiPure Universal RNA kit (Magen, Guangzhou, China) following the manufacturer’s instructions. The first strand cDNAs were synthesized from 1 μg of total RNA using a ReverTra Ace® quantitative PCR (qPCR) RT Master mix with gDNA Remover (Cat#: FSQ-301, ToYoBo, Japan) according to the manufacturer’s protocol. The cDNA samples were diluted to 200 ng for quantitative real-time PCR analyses. The primers were for mouse *Fmr1* (5′-GACAAGTCAGGAGTTGTGAGG-3′ and 5′-CTTTAAATAGTTCAGGTGATAATC-3′) and housekeeping gene *β-actin* (5′-TGGTCGTCGACAACGGCTC-3′ and 5′-CCATGTCGTCCCAGTTGGTAAC-3′). PCR experiments were performed using Dream Taq PCR Master Mix (Cat#: K1081, Thermo Scientific) following the cycling reactions. The qPCR experiments were performed on Rotor-Gene^TM^ Q instrument (QIAGEN, USA) according to the manufacturer’s instructions, and the relative cycle threshold (CT) values were normalized by *β-actin*.

### Western blotting and co-immunoprecipitation

The striatal tissues of WT and P608L mutation mice were prepared in RIPA (Radio Immunoprecipitation Assay) buffer (Millipore) on ice for 5 min (1 ml RIPA buffer per 1 ml cells pellet), followed by centrifuging at 13 000 × *g* for 5 min. Mice brain proteins were quantified using BCA (Bicin-choninic Acid) method. Western blots and immunoprecipitation procedures were performed as previous described.^34,35^ The antibodies used include DOPA decarboxylase (DDC), TH, monoamine oxidase B (MAO-B), dopamine transporter (DAT), vesicular monoamine transporter (VMAT2), Na-K-ATPase, extracellular signal–regulated kinase (ERK), p-ERK, G protein-coupled receptor kinase 2 (GRK2), D1R and Glyceraldehyde-3-phosphate dehydrogenase (GAPDH). The densities of the immunoreactive bands were determined by the software ImageJ.

### Liquid chromatography with tandem mass spectrometry analysis

The liquid chromatography with tandem mass spectrometry (LC-MS/MS) analysis and protein identification were conducted as previously described, with minor modifications.^34^ In brief, striatum samples were frozen in liquid nitrogen and subsequently stored at −80°C for further analyses. Six samples (three mice per group) were utilized for the proteomic analyses. Tissues were pulverized and homogenized using an SDT solution (4% SDS, 100 mM DTT, 100 mM Tris/HCl, pH7.6). Protein quantification was performed using the BCA method, followed by digestion with trypsin and labelling with the TMT Mass Tagging kit (Thermo Fisher Scientific, Rockford, IL, USA) in accordance with the manufacturer’s instructions. The LC-MS/MS spectral data were searched against the mouse FASTA database from UniProt using Proteome Discoverer 1.4 software (Thermo Scientific) at a false discovery rate of ≤0.01. For protein quantification, protein ratios were calculated as the median for unique peptides corresponding to each protein. Experimental bias was addressed by normalizing all peptide ratios to the median protein ratio. A global heat map analysis was performed to illustrate relative changes in protein levels at the proteome level in each group, based on hierarchical clustering analysis.

### Measurements of protein kinase A activity

PKA kinase activity was determined using a PKA Kinase Activity Assay kit (ab139435, Abcam, UK). Tissues were homogenized in a buffer containing 50 mM Tris-HCl (at pH 7.4), 5 mM MgCl_2_, 1 mM DTT and 10% glycerol, protease inhibitor cocktail and 1 mM PMSF (Phenylmethylsulfonyl fluoride) and were centrifuged at 20 000 × *g* at 4°C for 15 min. The supernatant was normalized for protein concentration and diluted 10-fold for detection. All the samples were detected according to the protocols. The optical densities of the wells were read on a microplate reader at 450 nm^36^

### Treatment with dopamine D1 receptor agonist SKF81297

The patient with the *FMR1* missense mutation exhibits poor sensitivity to L-dopa but demonstrates effective therapeutic response to D1R agonists for motor recovery. To investigate the effects of varying doses of the D1R agonist SKF81297, the compound was dissolved in saline (0.9% NaCl) to achieve injectable volumes of 0.08 mg/kg and 0.2 mg/kg. In each experiment, either the vehicle (saline) or SKF81297 (at a dose of 0.08 or 0.2 mg/kg) was administered subcutaneously (s.c.) for 15 min prior to the conditioning stage of the behavioural tests.

### Statistical analysis

All numerical data were expressed as mean ± SEM or mean ± SD. Statistical difference between two groups was determined by unpaired Student’s *t*-test. The value of <0.05 was considered be statistical difference. All statistical analyses were performed with SPSS (version 22.0) software.

## Results

### Characteristics of this patient with parkinsonism

**Figure 1:**
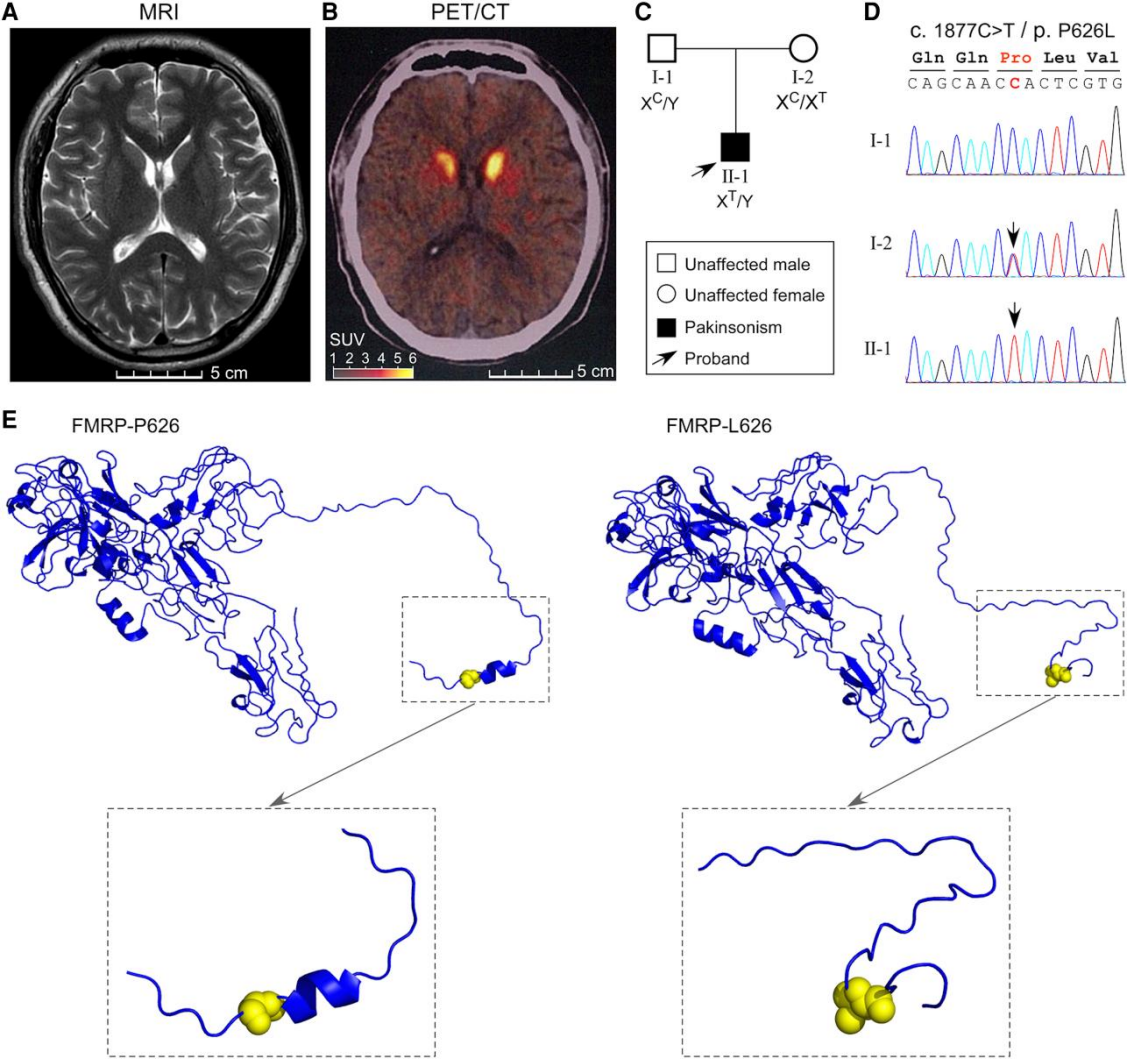
**Brain imaging and FMRP-P626L mutation characterization of a male patient with parkinsonism.** (**A**) A representative T2-weighted MRI scanning image of the patient. His MRI scanning was normal. (**B**) A representative PET/CT image of the patient. His PET/CT scanning indicated reduced DA transporter in the bilateral putamen. (**C**) Identification of a missense mutation c.1877C>T (p.P626L) in the *FMR1* gene from the patient, which is derived from his mother. (**D**) PCR and sequencing confirmation of the FMRP-P626L mutation from the lymphocyte-derived DNA of this family. The arrows show this mutation in the patient and his mother. (**E**) Three-dimensional (3D) protein structure prediction of the WT FMRP (FMRP-P626) and its mutant (FMRP-L626) using I-TASSER platform. The 3D structure of FMRP-L626 C terminus differs from FMRP-P626 C terminus. SUV, standardized uptake value.

The patient is a 34-year-old man who presented a history of progressive rigidity and bradykinesia lasting more than four years. He experienced the onset of left leg stiffness and gait slowing at the age of 30 years old. After 6 months, the stiffness progressed to his left upper limbs, accompanied by slow movement. Over the next 2 years, the symptoms extended to the right side, along with increasing irritability, depression, erectile dysfunction and nocturia. There was no family history of similar disorders or mental retardation. Upon examination, the patient exhibited normal cognitive function but showed rigidity in both extremities and postural tremors in his arms. The patient had a normal-appearing MRI scan (Fig. 1A). However, his PET/CT imaging indicated reduced DA transporter in the bilateral putamen (Fig. 1B). In terms of treatment, the patient was found to be insensitive to L-dopa, but exhibited a significant response to pramipexole and piribedil.

### A novel mutation P626L in the *FMR1* gene of the patient

Genetic analysis of the *FMR1* gene, suggested as one of the potential causes of parkinsonism, revealed a normal 27 CGG repeats in the 5’ UTR region; however, a missense mutation at the coding region (NM_002024.6: c.1877C>T, p.P626L) was identified in both the patient and his mother (Fig. 1C and D). The sequencing analysis revealed that the *FMR1* variant is located on the X chromosome of the mother, presenting as a heterozygous mutation without associated disease.

Sequencing analysis of those genes (including parkin/PARK2, PINK1 and DJ1/PARK7, as well as the common dystonia gene DYT1) associated with autosomal recessive Parkinson’s disease or parkinsonism did not identify any pathogenic variants, aside from some SNPs (Single nucleotide polymorphisms) noted in three databases (LOVD Parkinson’s Disease Mutation Database, Human Gene Mutation Database and SNP Database).

### A potential pathogenic effect of the FMRP P626L variant

The pathogenicity of the missense variant *FMR1* (c.1877C>T, p.P626L) was predicted by several *silico* tools, including SIFT, Polyphen-2, Mutation Master, LRT, VEST3, M-CAP, CADD and DANN. All these tools indicated that this variant is a damaging mutation (Supplementary Table 1). The allele frequency of this variant is absent in the general population (Supplementary Table 2). To predict the potential impact of the mutant on protein structure, we utilized I-TASSER for structural prediction. The results indicated that an α-helix is present near residue 626 at the C-terminal of wild-type FMRP, whereas no secondary structure was observed at the C-terminal of the FMRP-P626L mutation (Fig. 1E). This variant may affect protein function by interrupting spatial structure.

### Age-dependent motor deficits of the FMRP-P608L mutation mice

**Figure 2:**
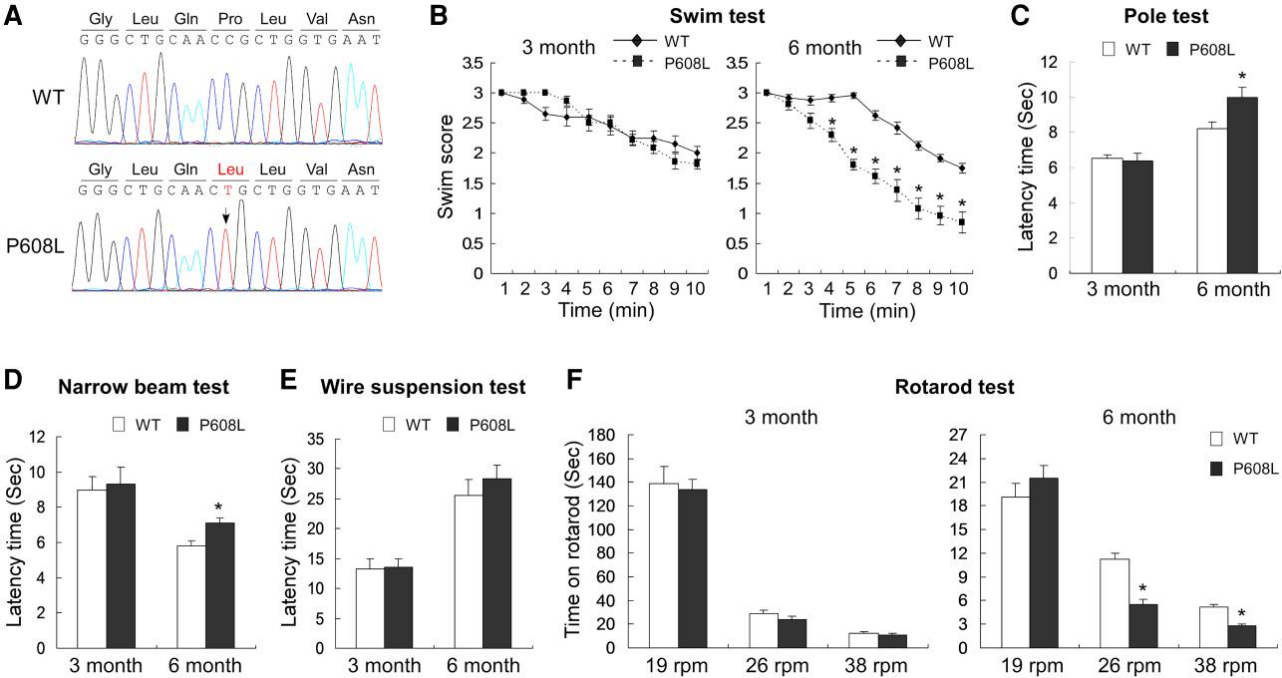
**Identification of the effect of an FMRP-P608L (matched with human P626L) mutation on motor behaviours by generating a transgenic mouse model.** (**A**) PCR and sequencing confirmation of the *Fmr1* missense mutation (FMRP-P608L) in the transgenic mice. The arrow shows the mutation site. (**B**) Swim tests showing a significant decrease in swim score of the P608L mutation mice compared with the WT mice at the age of 6 months old from 4-min time point (*t* = 5.3, df = 22, *P < 0.0001, unpaired *t*-test) to 10-min time point (*t* = 5.3, df = 22, *P < 0.0001, unpaired *t*-test), but no difference at the age of 3 months old (4-min: *t* = 0.8, df = 22, *P* = 0.444, unpaired *t*-test; 10-min: *t* = 1.5, df = 22, *P* = 0.152, unpaired *t*-test). (**C**) Pole tests showing a significant increase in latency time of the P608L mice compared with the WT mice at the age of 6 months old (*t* = 3.8, df = 22, *P = 0.0004, unpaired *t*-test), but no difference at the age of 3 months old (*t* = 0.3, df = 22, *P* = 0.734, unpaired *t*-test). (**D**) Narrow beam tests showing a significant increase in latency time of the P608L mice compared with the WT mice at the age of 6 months old (*t* = 4.2, df = 22, *P = 0.0003, unpaired *t*-test), but no difference at the age of 3 months old (*t* = 1.0, df = 22, *P* = 0.324, unpaired *t*-test). (**E**) Wire suspension tests showing no difference in latency time at the age of both 3 months old (*t* = 0.1, df = 22, *P* = 0.896, unpaired *t*-test) and 6 months old (*t* = 1.1, df = 22, *P* = 0.271, unpaired *t*-test). (**F**) Rotarod tests showing a significant decrease in the time staying on rotarod of the 6-month P608L mutation mice compared with the age-matched WT mice at 26-rpm rotation and 38-rpm rotation (19 rpm: *t* = 1.0, df = 22, *P* = 0.338; 26 rpm: *t* = 5.8, df = 22, *P < 0.0001; 38 rpm: *t* = 5.6, df = 22, *P < 0.0001; unpaired *t*-test), but no difference at the age of 3 months old (19 rpm: *t* = 0.3, df = 22, *P* = 0.759; 26 rpm: *t* = 1.5, df = 22, *P* = 0.184; 38 rpm: *t* = 0.6, df = 22, *P* = 0.588; unpaired *t*-test). Each data point or column shown as mean ± SEM represents the values from 12 animals in each data group.

To elucidate the pathophysiological consequences of the FMRP-P626L mutation, we generated a mouse model with a FMRP-P608L mutant matched with human FMRP-P626L mutant, which was confirmed by sequencing of the PCR product from the genomic region containing the anticipated mutation site (Fig. 2A). Given that the patient exhibited a parkinsonian phenotype, we propose that this mutant may lead to similar behavioural deficiencies in mouse models. To identify the phenotype associated with this variant, several motor behaviours were selected for testing this hypothesis. Compared with the 6-month-old WT mice, the 6-month-old P608L mice tended to show a lower swim score during the final seven minutes in swim test, although no significant changes were noted during the initial three minutes (Fig. 2B). Additionally, the 6-month-old P608L mice exhibited an increased latency in both the pole test and narrow beam test compared with the age-matched WT mice (Fig. 2C and D). However, no significant difference was observed between the two groups in the wire suspension test (Fig. 2E). The 6-month-old P608L mice exhibited less time on the apparatus at 26 and 38 rpm compared with the WT mice, while performance at 19 rpm remained unchanged between the two groups in the rotarod test (Fig. 2F). Importantly, there were no differences in any of the aforementioned motor behaviours between 3-month-old WT and P608L mice. These behavioural tests suggest that FMRP-P608L mice may exhibit age-related motor dysfunctions. In addition, we assessed the impact of this variant on memory behaviours. The alteration ratio of P608L mice remained unchanged compared with WT mice in the Y-maze test (Supplementary Fig. 1A). Moreover, there were no significant differences in error rates and latency between WT and P608L mice in the step-down test (Supplementary Fig. 1B). These findings suggest that this mutant does not lead to impairments in cognitive phenotypes.

### The content of dopamine is decreased in the FMRP-P608L mice

**Figure 3:**
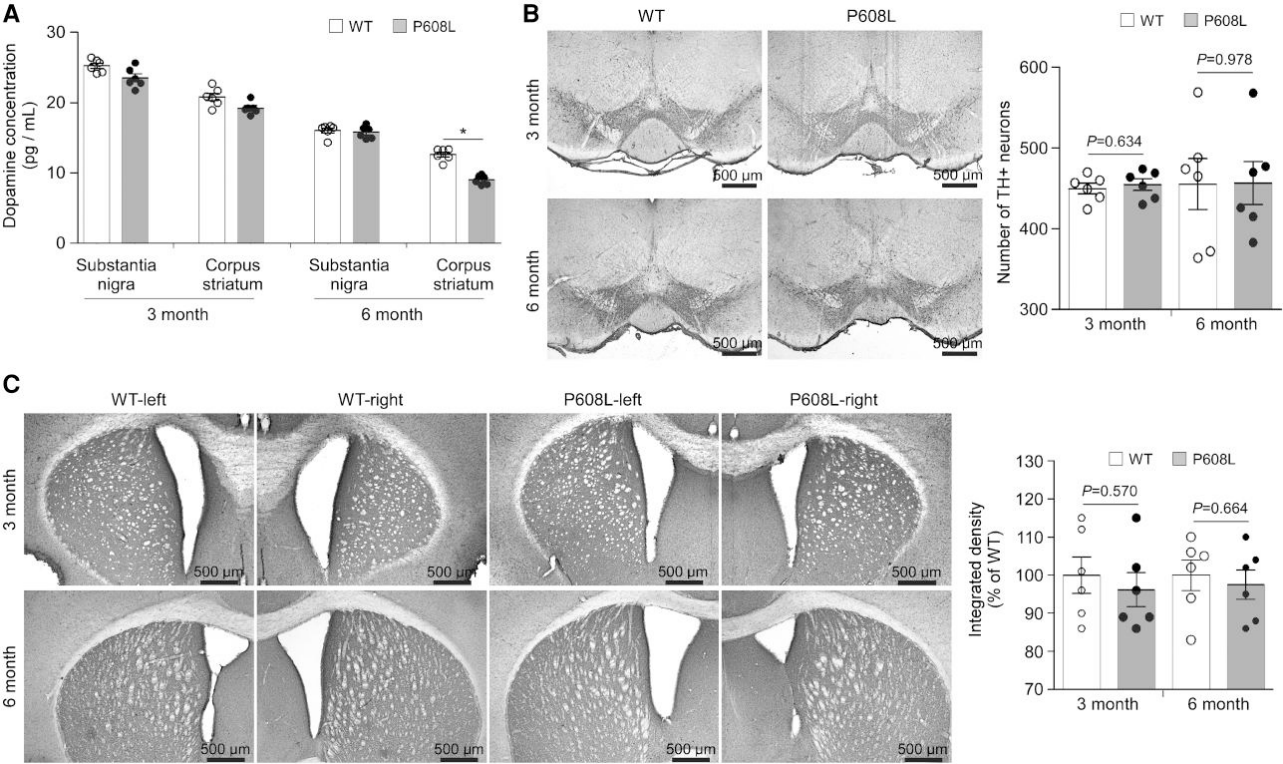
**Comparison of the DA content and TH-positive (TH^+^) neurons of the P608 mice against the WT mice.** (**A**) No alteration in DA content was seen in the substantia nigra and corpus striatum between the two groups at the age of 3 months old (nigra: *t* = 2.0, df = 10, *P* = 0.072; striatum: *t* = 2.2, df = 10, *P* = 0.051; unpaired *t*-test), as well as in the nigra between the two groups at the age of 6 months old (*t* = 0.5, df = 10, *P* = 0.608; unpaired *t*-test). The DA contents in the striatum of the P608L mice were lower than that of the WT mice (*t* = 8.2, df = 10, *P < 0.0001; unpaired *t*-test). (**B**) Immunohistochemistry showed that no change of the number of TH^+^ neurons was observed in the substantia nigra between the mutation and WT groups at both 3 and 6 months old (3 month: (*t* = 0.5, df = 10; 6 month: *t* = 0.1, df = 10; unpaired *t*-test). (**C**) No alteration in the integrated density of corpus striatum between the two groups at both 3 and 6 months old (3 month (*t* = 0.6, df = 10; 6 month: *t* = 0.4, df = 10; unpaired *t*-test). Each data point or column shown as mean ± SEM represents the values from six animals.

**Figure 4:**
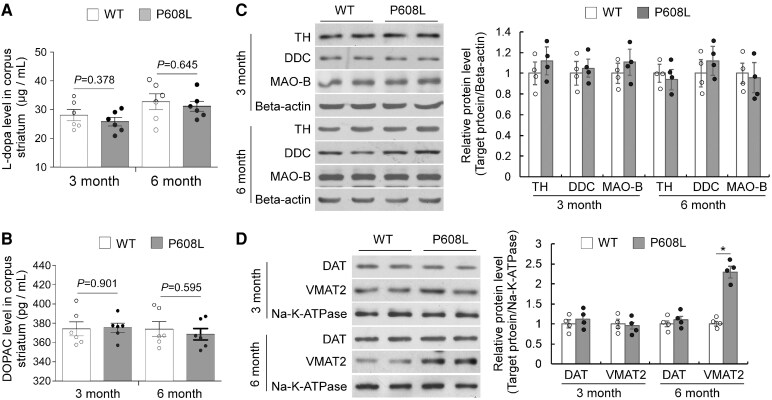
**Comparison of some metabolites and several proteins associated with DA synthesis, metabolism and transport in the striatum of the P608L mice against the WT mice.** (**A** and **B**) No significant difference in the concentrations of striatal L-dopa and DOPAC was detected between the mutation and WT mice at the age of both 3 and 6 months old (L-dopa/3 month: *t* = 0.9, df = 10; L-dopa/6 month: *t* = 0.5, df = 10; DOPAC/3 month: *t* = 0.1, df = 10; DOPAC/6 month: *t* = 0.5, df = 10; unpaired *t*-test). Each data column shown as mean ± SEM represents the values from six animals. (**C**) No significant difference in the relative protein levels of TH, DDC and MAO-B was found between the P608L mutation and WT mice at both 3 and 6 months of age (TH/3 month: *t* = 1.1, df = 6, *P* = 0.299; DDC/3 month: *t* = 0.6, df = 6, *P* = 0.572; MAO-B/3 month: *t* = 0.8, df = 6, *P* = 0.458; TH/6 month: *t* = 0.4, df = 6, *P* = 0.698; DDC/6 month: *t* = 1.0, df = 6, *P* = 0.348; MAO-B/6 month: *t* = 0.3, df = 6, *P* = 0.783; unpaired *t*-test). Each data point/column shown as mean ± SEM represents the values from four animals. (**D**) No significant change in the relative membrane protein levels of striatal DAT and VMAT2 were detected between the mutation and WT mice at 3 months of age (DAT: *t* = 0.7, df = 6, *P* = 0.496; VMAT2: *t* = 0.4, df = 6, *P* = 0.701; unpaired *t*-test). At 6 months of age, the levels of VMAT2, instead of DAT, significantly increased in the mutation mice compared with that of the WT mice (DAT: *t* = 0.4, df = 6, *P* = 0.696; VMAT2: *t* = 10.7, df = 6, *P < 0.0001; unpaired *t*-test). Each data point/column shown as mean ± SD represents the values from four animals.

To determine the relationship between DA’s actions and bradykinesia, we examined some indicators regarding DA synthesis, metabolism and transport in WT and FMRP-P608L mice. We found that the DA concentrations in the striatum of 6-month-old P608L mice were significantly decreased compared with that of the WT mice; however, no alterations were observed in the substantia nigra (Fig. 3A). Notably, no significant difference in the DA level was found in both the striatum and substantia nigra of the 3-month-old P608L mice compared with the age-matched WT mice (Fig. 3A). The densities of striatal DA fibres and the number of dopaminergic neurons in the substantia nigra remained unchanged in both WT and P608L mice at ages of 3 and 6 months (Fig. 3B and C). These results suggest that the reduction of striatal DA levels occurred in an age-dependent manner, which was not associated with the number of DA neurons. In addition, the levels of L-dopa, DOPAC, and the expression of TH, DDC, MAO-B, and DAT in the striatum of P608L mice at both 3 and 6 months of age did not differ from those in the WT group (Fig. 4A–C). Only the levels of vesicular monoamine transporter type 2 (VMAT2) on the membrane of 6-month-old P608L mice were significantly increased compared with the WT mice, while no changes were observed in the 3-month-old P608L mice (Fig. 4D). Taken together, these data suggest an age-dependent decrease in striatal DA of the P608L mice, which might be result from upregulation of VMAT2 on the membrane that could promote DA export.

### D1 receptor signalling is impaired in the FMRP-P608L mice

**Figure 5:**
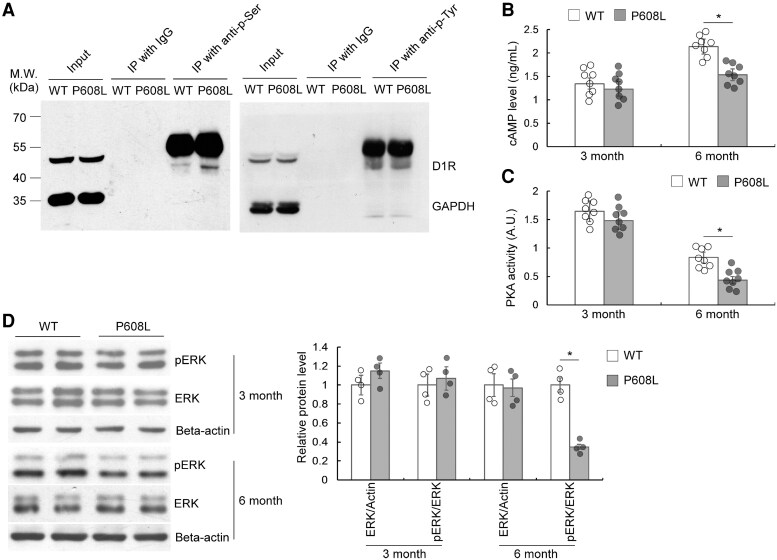
**Age-dependent impairment of striatal D1R signalling pathway in FMRP-P608L mutation mice.** (**A**) Western blotting with striatal Co-IP precipitates from 6-month-old mice showing that D1 receptor tended to be phosphorylated at serine residues (p-Ser) in the P608L mutation mice, instead of tyrosine residues (p-Tyr). (**B**) ELISA analyses showing a decrease of the striatal cAMP levels in the mutation mice at 6 months old (*t* = 5.7, df = 14, *P < 0.0001; unpaired *t*-test), but not at 3 months old (*t* = 0.6, df = 14, *P* = 0.575; unpaired *t*-test). Each data point/column shown as mean ± SD represents the values from eight animals. (**C**) ELISA analyses showing a decrease of the striatal PKA activities in the mutation mice at 6 months old (*t* = 5.1, df = 14, *P = 0.0002; unpaired *t*-test), but not at 3 months old (*t* = 0.6, df = 14, *P* = 0.571; unpaired *t*-test). Each data point/column shown as mean ± SD represents the values from eight animals. (**D**) Western blotting showing that the relative levels of striatal pERK/ERK were decreased in the P608L mice at 6 months of age compared with the age-matched WT mice (*t* = 6.6, df = 6, *P = 0.0006; unpaired *t*-test), but not at 3 months of age (*t* = 0.3, df = 6, *P* = 0.808; unpaired *t*-test). Each data point/column shown as mean ± SD represents the values from four animals.

To further confirm the alteration of DA signal in the FMRP-P608L mutation, co-immunoprecipitation (Co-IP) showed that D1 receptor was phosphorylated at serine residue rather than tyrosine residue in the 6-month-old FMRP-P608L mice (Fig. 5A). The ELISA data indicated that levels of cAMP and PKA activity decreased in the 6-month-old P608L mice, suggesting that the FMRP-P608L mutation contributes to the impairment of the D1 receptor through the PKA signalling pathway (Fig. 5B and C). Additionally, western blot demonstrated a reduction in the phosphorylation level of extracellular signal–regulated kinase (ERK) in the 6-month-old P608L mice. Notably, there were no significant differences observed between the 3-month-old P608L mice and WT mice (Fig. 5D). These results suggest that the P608L mutation induces an abnormal DA signalling pathway in an age-dependent manner.

### Age-dependent increased interaction between FMRP-P608L mutation and G protein–coupled receptor kinase 2

Given that the major function of FMRP is control of its target gene expression at translational level, we first used a TMT-based LC-MS/MS analysis to determine whether the P608L mutation affects the FMRP’s function in the control of gene expression. We found that only 40 proteins (17 upregulated and 23 downregulated) were changed with no more than 2-folds in the stratum of FMRP-P608L mice compared with the WT mice (Supplementary Tables 3 and 4), suggesting a mild alteration of the striatal proteome in the FMRP-P608L mice. Therefore, we hypothesize this FMRP mutant may impair the D1R signalling by affecting the interaction between FMRP and other proteins.

**Figure 6:**
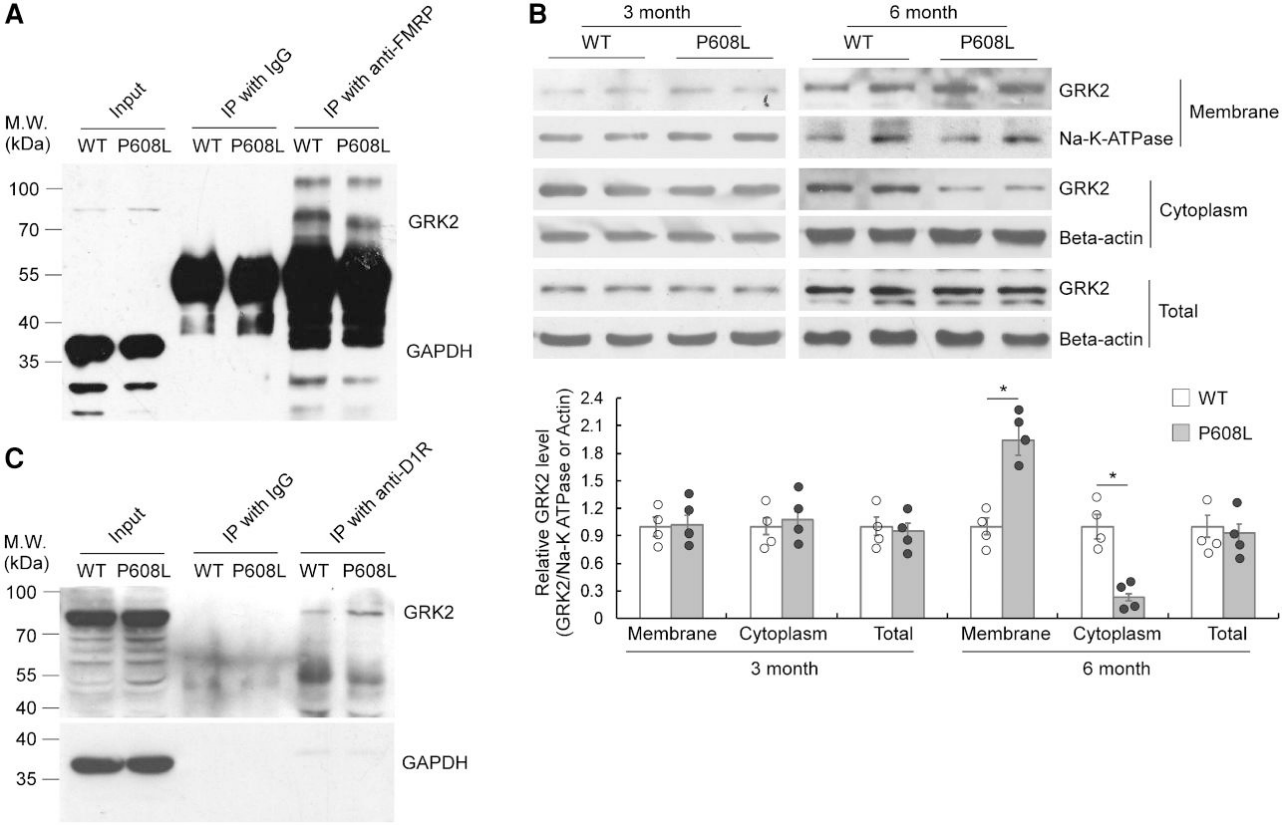
**Altered interaction among FMRP-P608L mutant, GRK2 and D1R.** (**A**) Western blotting with the striatal samples immunoprecipitated by anti-FMRP from 6-month-old mice showing an obvious decrease in the binding ability between the FMRP-P608L mutant and GRK2. (**B**) Western blotting showing that no alteration of relative striatal GRK2 levels in the membrane, cytoplasm and total protein extracts between the P608L mutation and WT mice at the age of 3 months (membrane: *t* = 0.4, df = 6, *P* = 0.692; cytoplasm: *t* = 1.1, df = 6, *P* = 0.338; total: *t* = 0.5, df = 6, *P* = 0.665; unpaired *t*-test). The relative GRK2 levels in the membrane and cytoplasm of the mutation mice were respectively significantly higher and lower than that of the WT mice at the age of 6 months, but no alteration in the total protein extracts (membrane: *t* = 8.6, df = 6, *P = 0.0001; cytoplasm: *t* = 6.7, df = 6, *P = 0.0006; total: *t* = 0.4, df = 6, *P* = 0.721; unpaired *t*-test). Each data point/column shown as mean ± SD represents the values from four animals. (**C**) Western blotting with the striatal samples immunoprecipitated by anti-D1R from 6-month-old mice showing an obvious increase in the binding ability between the GRK2 mutant and D1R.

We then predict whether the FMRP-P608L mutation influences the binding capability of FMRP to other proteins. IPred2A software prediction showed a decrease in the C-terminal binding protein ability of the FMRP-P608L (Supplementary Fig. 2). A previous research has established that FMRP serves as a crucial messenger for DA signalling by interacting with GRK2 through protein–protein interaction, and the absence of FMRP leads to the redistribution of GRK2.^18^ Co-IP was conducted to determine whether the interaction between FMRP and GRK2 is affected by the FMRP-P608L mutation. The results revealed a reduced binding affinity of FMRP to GRK2 in 6-month-old P608L mice (Fig. 6A), suggesting that the impaired interaction between FMRP-P608L and GRK2 promotes the membrane localization of GRK2 (Fig. 6B). Furthermore, to elucidate the effects of GRK2 and D1 receptors on each other, we observed an increase in the binding of GRK2 to D1Rs in 6-month-old P608L mice (Fig. 6C). These findings indicate that the FMRP-P608L mutation may influence the D1 receptor through its interaction with GRK2.

### Alleviation of the motor deficits of the FMRP-P608L mice treated with a D1 receptor agonist

**Figure 7:**
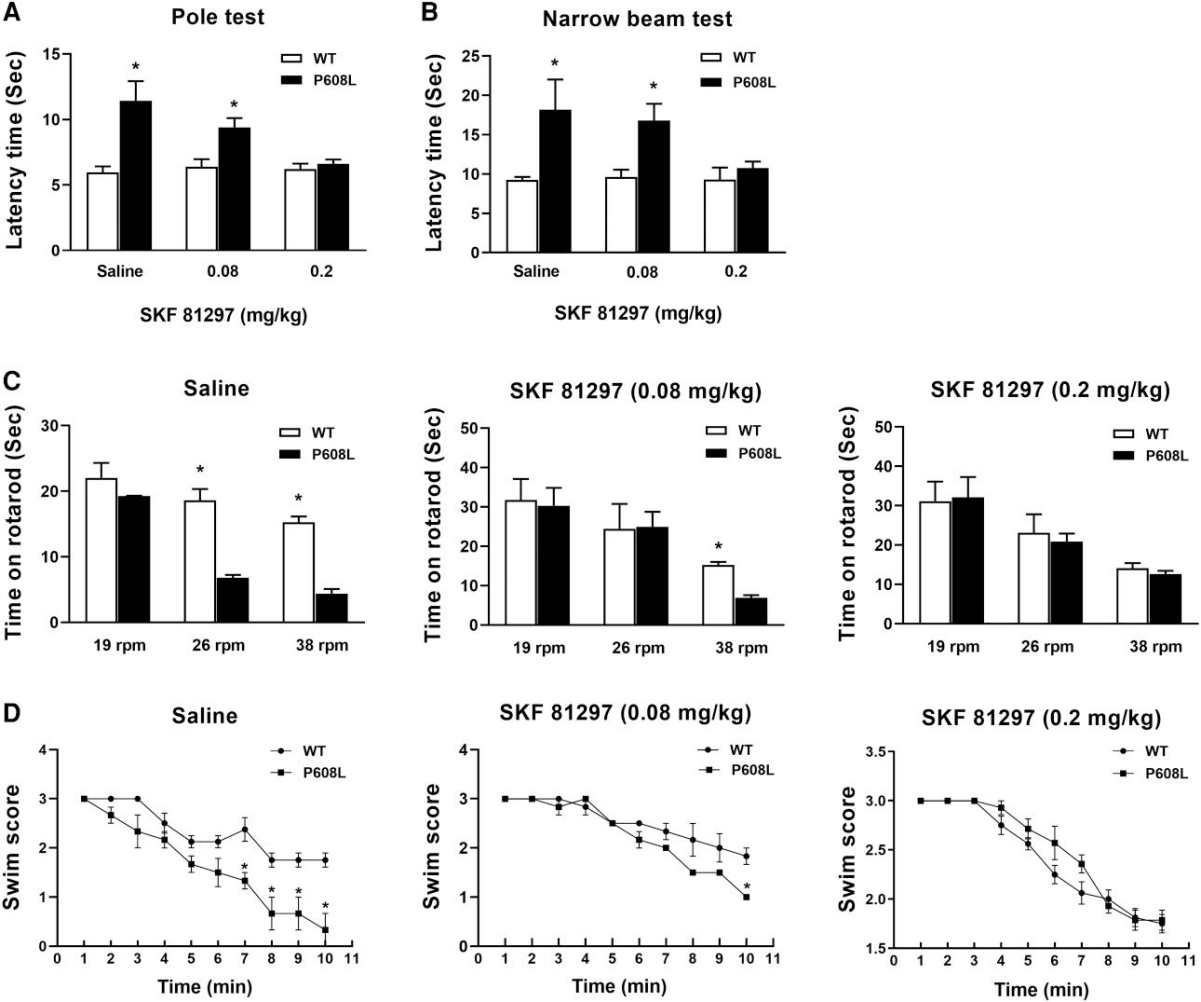
**The motor behaviours in 6 months old mutation and WT mice treated with diverse drug doze of SKF81297.** (**A**) Pole tests showing a significant increase in latency time of the P608L mice compared with the WT mice following treatment with saline (*t* = 4.054, df = 22, *P = 0.0098, unpaired *t*-test) and low-dose SKF81297 (*t* = 3.318, df = 22, *P = 0.0294, unpaired *t*-test), but no difference following treatment with high-dose SKF81297 (*t* = 0.7449, df = 22, *P* = 0.472, unpaired *t*-test). (**B**) Narrow beam tests showing a significant increase in latency time of the P608L mice compared with the WT mice following treatment with saline (*t* = 3.1, df = 22, *P = 0.0362, unpaired *t*-test) and low-dose SKF81297 (*t* = 2.759, df = 22, *P = 0.0399, unpaired *t*-test), but no difference following treatment with high-dose SKF81297 (*t* = 0.8366, df = 22, *P* = 0.4192, unpaired *t*-test). (**C**) Rotarod tests showing a significant decrease in the time staying on rotarod of the 6-month P608L mutation mice compared with the age-matched WT mice at 26-rpm rotation and 38-rpm rotation following treating with saline (19 rpm: *t* = 0.8103, df = 22, *P* = 0.4632; 26 rpm: *t* = 5.638, df = 22, *P = 0.0024; 38 rpm: *t* = 9.113, df = 22, *P = 0.0003; unpaired *t*-test) and a significant decrease in the time staying on rotarod of P608L mutation mice compared with the age-matched WT mice at 38-rpm rotation following treating with low-dose SKF81297 (19 rpm: *t* = 0.2099, df = 22, *P* = 0.844; 26 rpm: *t* = 0.0732, df = 22, *P* = 0.9462; 38 rpm: *t* = 8.436, df = 22, *P = 0.0011; unpaired *t*-test), but no difference at all rotation speeds treating with high-dose SKF81297 (19 rpm: *t* = 0.1298, df = 22, *P* = 0.8996; 26 rpm: *t* = 0.4716, df = 22, *P* = 0.6515; 38 rpm: *t* = 0.9366, df = 22, *P* = 0.3764; unpaired *t*-test). (**D**) Swim tests showing a significant decrease in swim score of the P608L mutation mice compared with the WT mice at the age of 6 months old from 7-min time point (*t* = 8.0, df = 22, *P = 0.015, unpaired *t*-test) to 10-min time point treating with saline (*t* = 4.243, df = 22, *P = 0.024, unpaired *t*-test) and decrease in swim score from 10-min time point treating with low-dose SKF81297 (6-min: *t* = 1.0, df = 22, *P* = 0.423, unpaired *t*-test; 9-min: *t* = 1.732, df = 22, *P* = 0.225, 10-min: *t* = 5.0, df = 22, *P = 0.038, unpaired *t*-test), but no difference in time point treating with high-dose SKF81297 (4-min: *t* = 1.472, df = 22, *P* = 0.1648, unpaired *t*-test; 10-min: *t* = 0.2582, df = 22, *P* = 0.8003, unpaired *t*-test). Each data point or column shown as mean ± SEM represents the values from 12 animals in each data group.

It has been reported that the binding of GRK2 to D1 receptors leads to the phosphorylation of D1 receptors.^18^ Given that this patient is particularly sensitive to the efficacy of D1 receptor agonists, we aim to evaluate the effectiveness of a D1R agonist SFK81297 in the treatment of movement disorders. The WT and P608L mice were respectively administered with saline, a low dose (0.08 mg/kg) and a high dose (0.2 mg/kg) of SKF81297 before motor behavioural tests. The results indicated no significant difference in latency time between WT and P608L mice in both the pole test (Fig. 7A) and the narrow beam test (Fig. 7B) following treatment with 0.2 mg/kg. Although the treated P608L mice exhibited poorer performance at speeds of 26 and 38 rpm when treated with saline, both WT and P608L mice exhibited similar performance times on the rotarod after receiving the 0.2 mg/kg dose (Fig. 7C). Furthermore, the swim scores of the P608L mice treated with 0.2 mg/kg did not differ from that of the WT group (Fig. 7D). Together, these data suggest that D1 receptor agonist can improve the motor abilities of P608L mice.

## Discussion

In this study, we report and characterize the effects of a novel *FMR1* missense mutation identified in a patient exhibiting typical FXTAS symptoms. The *FMR1* P626L mutation has been predicted to be a damaging variant that affects the protein binding ability at the C terminus of FMRP. To elucidate the molecular mechanisms underlying FXTAS associated with the *FMR1* P626L mutation, we generated and characterized a mouse model with the *Fmr1* P608L mutation. Our findings indicate that the *Fmr1* P608L mice exhibit motor deficits and reduced DA levels. Furthermore, our study demonstrates that the impaired interaction between FMRP-P608L and GRK2 facilitates the binding of GRK2 to D1R, leading to serine phosphorylation of the D1 receptor. This interaction inhibits the cAMP/PKA pathway, consequently decreasing DA efficiency. Notably, the motor disabilities of the FMRP-P608L mice were alleviated through treatment with a D1R agonist. To our knowledge, this C terminus missense mutation represents the firstly identified alteration in FMRP function associated with parkinsonism.

FXTAS is characterized by classical clinical manifestations including motor dysfunction, cognitive decline and parkinsonism.^7,37^ Some studies have reported that the MRI images of some FXTAS patients exhibited white matter hyper-intensities and atrophied grey matter linked to age-dependent in cognitive deficits.^38^ However, the onset age of the patient identified in this study was 34 years old, and his MRI images did not show white matter hyper-intensities. Furthermore, no cognitive disability of the patient was observed, which is consistent with the P608L mutation mouse model. In addition, abnormalities of white matter of those FXTAS patients were identified to be strongly correlated with the increase in *FMR1* mRNA level.^39,40^ In our study, no significant change of the *Fmr1* mRNA levels was found in the FMRP-P608L mice, which may also be one of the reasons that the MRI scans of this patient did not exhibit abnormalities. The majority of patients exhibit a mixed movement disorder, presenting with signs of bradykinesia, tremor, ataxia and parkinsonism.^41^ In our study, the patient with the *FMR1* variant displayed a phenotype of motor impairment, likely caused by genetic mutations that contribute to pathogenesis as predicted by analytical tools. This finding suggests that the patient may exhibit FXTAS-like parkinsonism due to point mutations. Similar phenotypes have been observed in FXTAS animal models; however, motor abilities vary across different mouse models.^42^ In the rotarod test, an age-dependent decline in motor performance was observed in the expanded CGG KI Dutch model at 20, 52 and 72 weeks of age.^43^ However, no differences in rotarod performance were observed between the NIH mice and WT animals.^44^ In our study, we identified that the FMRP-P608L mice exhibited an age-related motor deficit in the rotarod test, pole test, narrow beam test and swim test. Previous research have demonstrated that point mutations of the *FMR1* gene are associated with FXS pathogenesis by affecting the function of FMRP.^12^ To our knowledge, there is no report on the relationship between *FMR1* missense mutations and FXTAS, thus this study provides a new contribution of *FMR1* missense mutation, to parkinsonism-like phenotypes.

It is well known that motor impairment is associated with DA deficiency. FMRP is involved in several post-synaptic signalling cascades, including those related to acetylcholine, DA and glutamate receptor signalling.^18,45,46^ Dysregulation of the DA pathway due to FMRP dysfunction may contribute to some phenotypes of those *FMR1*-related disorders. Some FXTAS patients exhibit DA deficiency alongside motor dysfunction and cognitive decline.^47^ Furthermore, the motor dysregulation and cognitive deficits observed in *Fmr1*-KO mice suggest that dysfunctions in corticostriatal systems may contribute to these deficits through a reduction in dopaminergic stimulation, resulting in an imbalance in DA release.^48^ It has been established that a decrease in dopaminergic neurons or impairment of the nigra striatal pathway leads to a reduction in DA content, which subsequently results in motor deficiency.^49^ Our results indicate that, although there is an age-related decline in intracellular DA concentration in P608L mice, no differences were found in dopaminergic neurons or striatal DA fibres. The present study has shown no changes in the indicators of DA-related proteins, DA synthesis, and metabolism between P608L and WT mice. These findings may support the hypothesis of decreased DA content affecting DA transmission. VMAT2 plays a crucial role in maintaining low levels of DA in the neuronal cytosol by sequestering DA into vesicles.^50^ A previous study has demonstrated disrupted vesicular storage of DA and decreased motor function in VMAT2-deficient mice.^51^ Additionally, age-related declines in striatal DA content and abnormal motor performance are observed in this mouse model.^52^ We further discovered an elevated level of VMAT2 membrane protein in the striatum of 6-month-old P608L mice, suggesting an increase in DA release from the cytoplasm, which may contribute to the decreased DA content.

FMRP is an RNA-binding protein that regulates various aspects of RNA biology, including RNA transport, stability and, most importantly, mRNA translation.^53,54^ However, the present study using LC-MS/MS analysis of FMRP-P608L mice suggested that this missense mutation may have minimal effects on the dysregulated expression of other proteins. Recent reports indicate that some missense mutations outside the 5′ UTR may also contribute to FXS by altering the function or structure of FMRP.^12,20,55^ Several animal models have shown that these variants may decrease FMRP expression or impair protein functions such as AMPA (A-amino-3-hydroxy-5-methyl-4-isoxazole-propionic acid) receptor transport, ribosomal translocation, synaptic plasticity, translation and even FMRP’s presynaptic function.^56-60^ Similarly, FMRP-R138Q mutation does not alter the protein expression of FMRP.^21^ This variant is associated with increased spine density, synaptic ultrastructural defects and elevated AMPA receptor surface expression. In our study, there were no significant differences in the levels of FMRP and *Fmr1* mRNA in the corpus striatum, and substantia nigra between the FMRP-P608L mice and WT group (Supplementary Fig. 3). Previous researches focused on the functions of the RNA-binding domains N-terminal domain of FMRP, NLS, KH1 and KH2, where missense mutations lead to decreased protein–protein interaction capabilities, resulting in nuclear mislocalization or impaired neural development.^17,20^ Furthermore, noncoding variants in the 5’ UTR and 3’ UTR hinder the initiation of *FMR1* transcription and gene expression respectively.^61^ An insertion mutation, c.1457insG/p.G538fs*23, was predicted to cause a frameshift that introduces 22 novel amino acid sequences resembling a motif of nuclear localization signals, which may lead to the abnormal localization of FMRP.^62^ Previous studies have demonstrated that several missense mutations at the N terminus of FMRP can reduce interactions with CYFIP1, FXR1 and 82-FIP, which in turn results in altered axonal and synaptic elaborations.^20,63,64^ Therefore, these findings implicate that the P608L mutation identified in this study may lead to dysfunction of FMRP by affecting its interaction with other proteins.

The C-terminal of FMRP plays a crucial role in nuclear localization signals and protein–protein interactions. A guanine insertion in *FMR1* exon 15 alters the localization and function of FMRP by creating a novel, short C-terminal sequence within the RGG box.^62^ Several FMRP target mRNAs have confirmed their binding to FMRP due to the presence of predicted G-quadruplex structures that interact with the C-terminal RGG box.^65^ Additionally, a new structure, SoSLIP, in *superoxide dismutase 1* mRNA may also interact with FMRP via the RGG domain.^66^ IUPred2A, which acts as a prediction of protein binding function,^27^ has shown a decreased interaction in C-terminal of FMRP-P608L. Furthermore, the C-terminal of FMRP interacts with Ran-binding protein in the microtubule-organizing centre, involving interacting partners for the D1 DA receptor.^19,67^ A previous study identified an interaction between FMRP and GRK2, which control the subcellular distribution of GRK2, and the absence of FMRP could lead to the redistribution of GRK2 in *Fmr1*-KO mice.^18^ The present study showed an age-related decline in the binding capability of GRK2 and FMRP-P608L in the striatum, where the GRK2 levels were increased in membrane preparations and decreased in cytosolic preparations from the striatum of 6-month-old P608L mice. These data suggest that FMRP acts as a key messenger for DA modulation by interacting with GRK2.

Previous evidence show that GRK2 is expressed in striatal neurons and acts as a regulator of DA receptor functions by phosphorylating active receptors such as D1R,^68^ and the phosphorylation of D1R occurs at either serine or tyrosine sites due to GRK2’s interaction with D1R.^69,70^ Another study has demonstrated that the membranous translocation of GRK2 increases serine phosphorylation of D1A receptors.^71^ The present study showed that the aggregation of membranous GRK2 integrated with D1R led to serine phosphorylation of D1R, while no tyrosine phosphorylation occurred in the context of the FMRP-P608L mutation. These results suggest that the FMRP-P608L mutation leads to hyper-phosphorylation of the D1R at serine sites by interaction with GRK2, which contributes to the impairment of D1R signalling in the striatum of *Fmr1*-KO mice.^18^ Typically, D1R signals through Gs to stimulate cAMP production, resulting in the activation of PKA and the modulation of neuronal activity.^72^ Importantly, GRK2 binds to and phosphorylates the D1 receptor, leading to a decrease in intracellular cAMP levels, which further contributes to the dysfunction of DA signalling.^73^ In our research, we discovered a reduced level of cAMP and decreased PKA activity in the striatum of 6-month-old P608L mice, indicating an abnormal cAMP/PKA signalling pathway. The stimulation of ERK1/2 is expected to activate various downstream targets and functional responses.^74^ The reduced cAMP production and inhibition of PKA activity in the striatum may lead to the dephosphorylation of ERK1/2 in 6-month-old P608L mice, potentially impairing DA downstream signalling and dopaminergic biological effects. FMRP has been shown to regulate PKA activity within the learning and memory circuitry of FXS models, suggesting that FMRP plays a role in the regulation of D1R through the PKA signalling pathway.^75^ A previous research has demonstrated that dopamine D1R agonist maintains motor coordination and balance in rats.^76^ SKF81297, acting as a D1R agonist, has been identified to alleviate dopaminergic motor dysfunctions in parkinsonian animals and patients.^77^ In our study, the motor dysfunctions in the FMRP-P608L mutation mice can be ameliorated by high doses of SKF81297, which should offer a potential therapeutic approach for parkinsonism patients, as well as FXTAS treatments.

## Conclusion

In summary, our data demonstrate that the *Fmr1* P608L variant impairs the downstream DA signalling pathway by reducing the interaction between FMRP and GRK2, which leads to age-dependent motor dysfunction in the mutant mice. Therefore, we speculate that the *FMR1* P626L variant identified in the patient may be implicated in the pathogenesis of symptoms analogous to FXTAS.

## Supplementary Material

fcaf338_Supplementary_Data

## Data Availability

The datasets generated and/or analysed during the current study are not publicly available due to part of the data in the study related to other studies but are available from the corresponding author on reasonable request. Original western blots in this study are shown in Supplementary Fig. 4.
