# Supplementary material for: Age-dependent impairment of dopamine D1 receptor signalling in mouse striatum by FMR1 variant P626L
Source: Brain Commun. 2025 Sep 10;7(5):fcaf338. doi: 10.1093/braincomms/fcaf338 (PMC12448934; doi:10.1093/braincomms/fcaf338)
Supplement: fcaf338_Supplementary_Data [file fcaf338_supplementary_data.pdf]

Supplementary Table 1 The pathogenicity of *FMR1* missense mutation (c.1877C>T) is predicted by *in-silico* tools.

Algorithm	Socre	prediction
SIFT	0.025	Damaging
Polyphen-2	0.962	Probably_damaging
Mutation Taster	1.000	Disease_causing
LRT	0.000	Deleterious
VEST3	0.755	Damaging
M-CAP	0.293	Damaging
CADD	22.7	Damaging
DANN	0.997	Damaging

Supplementary Table 2 The allele frequency in population of *FMR1* missense mutation (c.1877C>T) is predicted by *in-silico* tools.

**Dataset**	**Population**	**Allele frequency**
gnomAD_exome	All	-
gnomAD_exome	African American	-
gnomAD_exome	Latino	-
gnomAD_exome	Ashkenazi Jewish	-
gnomAD_exome	East Asian	-
gnomAD_exome	Finnish	-
gnomAD_exome	Non-Finnish European	-
gnomAD_exome	Other	-
gnomAD_exome	South Asian	-

Supplementary Table 3 The upregulated proteins in FMRP-P608L mice are measured by LC-MS/MS compared to WT mice.

Accession	Description	Coverage	Unique Peptides	Folds Exe/Con
A0A140T8J4	Heme-binding protein 1, GN=Hebp1	36.32	5	1.600073
P05784	Keratin, type I cytoskeletal 18, GN=Krt18	10.64	3	1.436672
Q9Z2K1	Keratin, type I cytoskeletal 16, GN=Krt16	5.76	1	1.411068
G3UZT4	Huntingtin-associated protein 1, GN=Hap1	3.87	1	1.354385
Q9D1R1	Complex I assembly factor TMEM126B, GN=Tmem126b	5.22	1	1.352951
Q91W06	V-type proton ATPase subunit a, GN=Tcirg1	0.84	1	1.350478
P11679	Keratin, type II cytoskeletal 8, GN=Krt8	11.22	2	1.300947
Q9R1E6	Ectonucleotide pyrophosphatase/phosphodiesterase family member 2, GN=Enpp2	1.39	1	1.283002
Q3THS8	Uncharacterized protein, GN=Rcl1	3.49	1	1.262634
D6RET7	GRAM domain-containing protein 4, GN=Gramd4	2.79	1	1.261937
Q5I0W5	Sdf2 protein, GN=Sdf2	4.49	1	1.244231
A0A0A6YX69	BTB/POZ domain-containing protein KCTD3, GN=Kctd3	6.2	1	1.229817
D3YZC2	Menin, GN=Men1	2.78	1	1.224493
Q149F3	Eukaryotic peptide chain release factor GTP-binding subunit ERF3B, GN=Gspt2	7.59	1	1.210187
Q05DI3	Stathmin, GN=Stmn4	14.77	2	1.207445
Q05BL6	Marcks protein (Fragment), GN=Marcks	22.82	2	1.204761
Q9CQ06	39S ribosomal protein L24, GN=Mrpl24	4.63	1	1.201893

17 upregulated proteins were found in the FMRP-P608L mice showed an increase of about 1.2-1.6 folds, but no more than 2 folds ($P<0.05$).

Supplementary Table 4 The downregulated proteins in FMRP-P608L mice are measured by LC-MS/MS compared to WT mice.

Accession	Description	Coverage	Unique Peptides	Folds Exe/Con
P46664	Adenylosuccinate synthetase isozyme 2, GN=Adss	26.32	8	0.614133
S4R2I0	F-box only protein 11 (Fragment), GN=Fbxo11	5.11	1	0.683722
Q9DB60	Prostamide/prostaglandin F synthase, GN=Fam213b	7.96	2	0.693933
V9GXC1	Peptidyl-prolyl cis-trans isomerase, GN=Gm12728	41.56	1	0.740173
Q8CEK6	Uncharacterized protein, GN=Shisa4	6.16	1	0.747878
H3BK16	ATP synthase mitochondrial F1 complex assembly factor 1, GN=Atpaf1	3.77	1	0.751122
Q8VED5	Keratin, type II cytoskeletal 79, GN=Krt79	6.78	1	0.763562
P14115	60S ribosomal protein L27a, GN=Rpl27a	25.68	4	0.766842
Q3TJZ7	Uncharacterized protein, GN=Jpt2	4.6	1	0.772453
F6ZIA4	Myelin basic protein (Fragment), GN=Mbp	80.89	2	0.790118
A2AIL4	NADH dehydrogenase (ubiquinone) complex I, assembly factor 6, GN=Ndufaf6	3.9	1	0.791688
Q8C1H7	Uncharacterized protein, GN=Ttc17	5.39	1	0.795712
P51910	Apolipoprotein D, GN=Apod	7.41	1	0.8025
A0A0U1RP60	CDP-diacylglycerol--inositol 3-phosphatidyltransferase, GN=Cdipt	6	1	0.816432
B1AXS4	Oligophrenin-1 (Fragment), GN=Ophn1	6.06	1	0.817752
Q8BL65	Actin-binding LIM protein 2, GN=Ablim2	22.88	1	0.818192
P17809	Solute carrier family 2, facilitated glucose transporter member 1, GN=Slc2a1	4.88	1	0.819404
Q3THU8	Uncharacterized protein, GN=Slc25a3	18.21	6	0.81956
Q8CHK3	Lysophospholipid acyltransferase 7, GN=Mboat7	2.54	1	0.822239
P02798	Metallothionein-2, GN=Mt2	34.43	3	0.822519
Q8JZR6	Electroneutral sodium bicarbonate exchanger 1, GN=Slc4a8	3.49	1	0.825378
G3XA25	Acetyl-CoA acetyltransferase, GN=Acat2	29.59	1	0.83041
Q6GQV8	Cytospin A, GN=Specc1l	3.6	2	0.832087

23 downregulated proteins were found in the FMRP-P608L mice showed an increase of about 0.61-0.83 folds, but no less than 0.5 folds ($P<0.05$).

Supplementary Figure 1

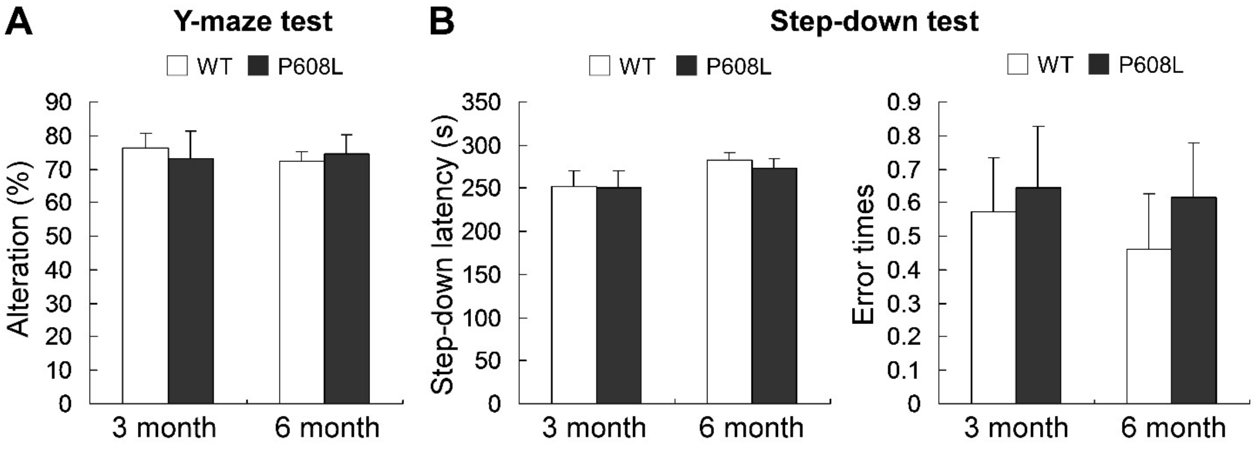

Supplementary Figure 1 Detection of memory ability of the FMRP P608L mutation and WT mice. (**A** and **B**) No significant change of alteration in Y-maze tests and latency and error times in step-down tests were observed between the FMRP P608L mice and the WT mice at the age of both 3 months and 6 months (alteration/3 month: t = 1.0, df = 22, P = 0.301; alteration/6 month: t = 0.1, df = 22, P = 0.924; latency/3 month: t = 0.4, df = 22, P = 0.669; latency/6 month: t = 0.1, df = 22, P = 0.896; error times/3 month: t = 0.4, df = 22, P = 0.704; error times/6 month: t = 0.3, df = 22, P = 0.296; unpaired t-test). Each data column shown as mean ± SEM represents the values from 12 animals.

Supplementary Figure 2

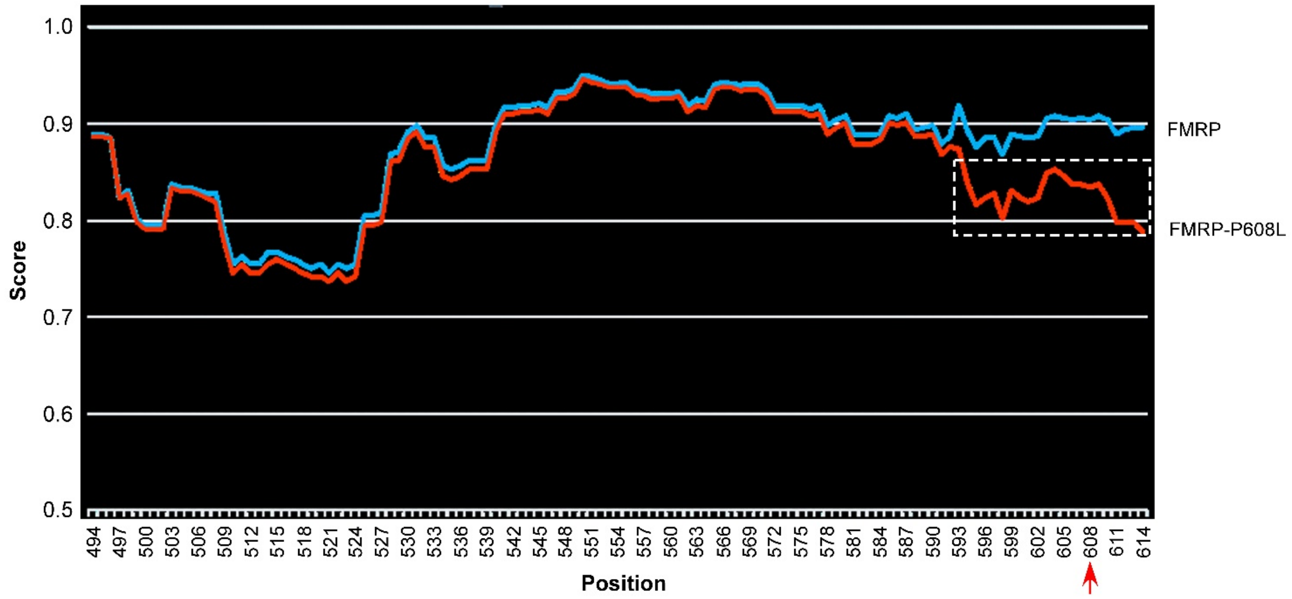

Supplementary Figure 2 Protein disorder prediction of FMRP and FMRP-P626L mutant by IUPred2A (Prediction of Intrinsically Unstructured Proteins). The C-terminals of both FMRP and FMRP-P608L mutant were predicted as disordered zones (score > 0.5), but the score of the C-terminal (aa 592-614) of FMRP-P608L was lower than that of the FMRP (dashed box).

Supplementary Figure 3

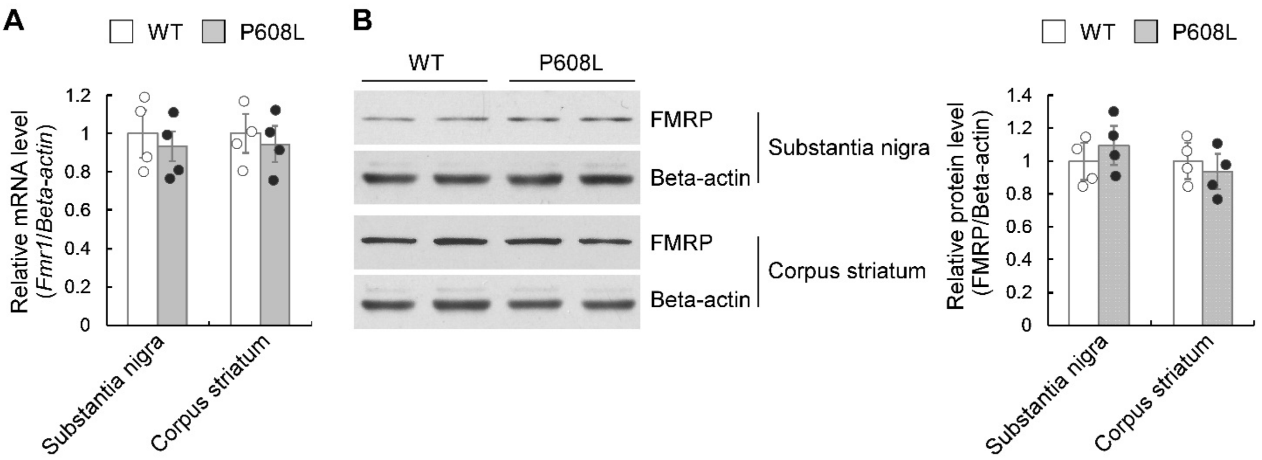

Supplementary Figure 3 Comparison of the *Fmr1* mRNA and FMRP levels in the substantia nigra and corpus striatum between FMRP P608L mutation and WT mice at the age of 6 months. (**A**) Real-time RT-qPCR assays showing no significant change in relative *Fmr1* mRNA levels in the nigra and striatum of FMRP P608L mutation mice compared to the WT mice (nigra: $t = 0.2$, df = 6, $P = 0.866$; striatum: $t = 0.2$, df = 6, $P = 0.813$; unpaired *t*-test). (**B**) Western blotting showing no significant difference in relative FMRP levels in the nigra and striatum between FMRP P608L mutation and WT mice (nigra: $t = 0.5$, df = 6, $P = 0.604$; striatum: $t = 0.6$, df = 6, $P = 0.576$; unpaired *t*-test). Each data point/column shown as mean ± SD represents the values from 4 animals.

Supplementary Figure 4

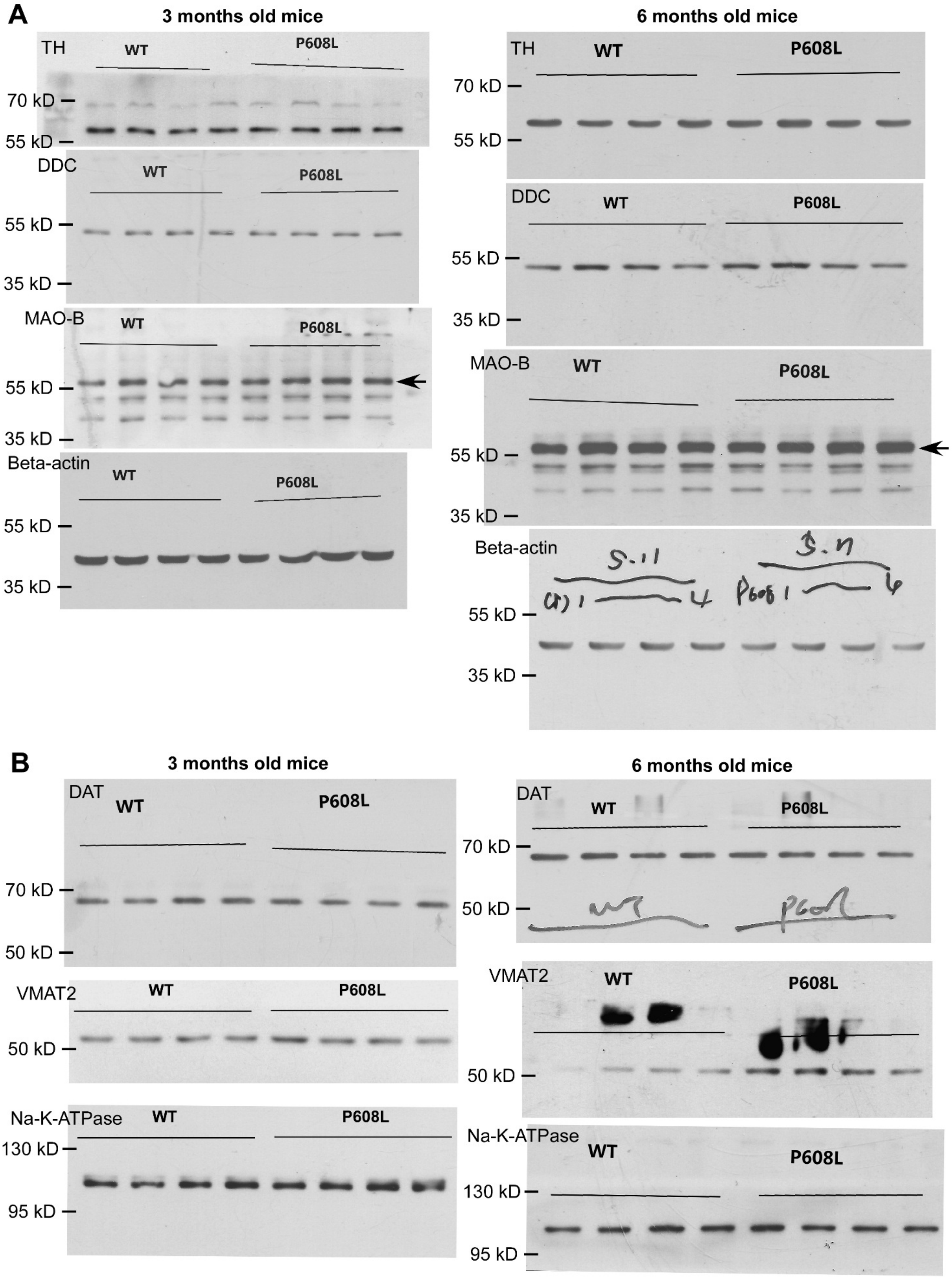

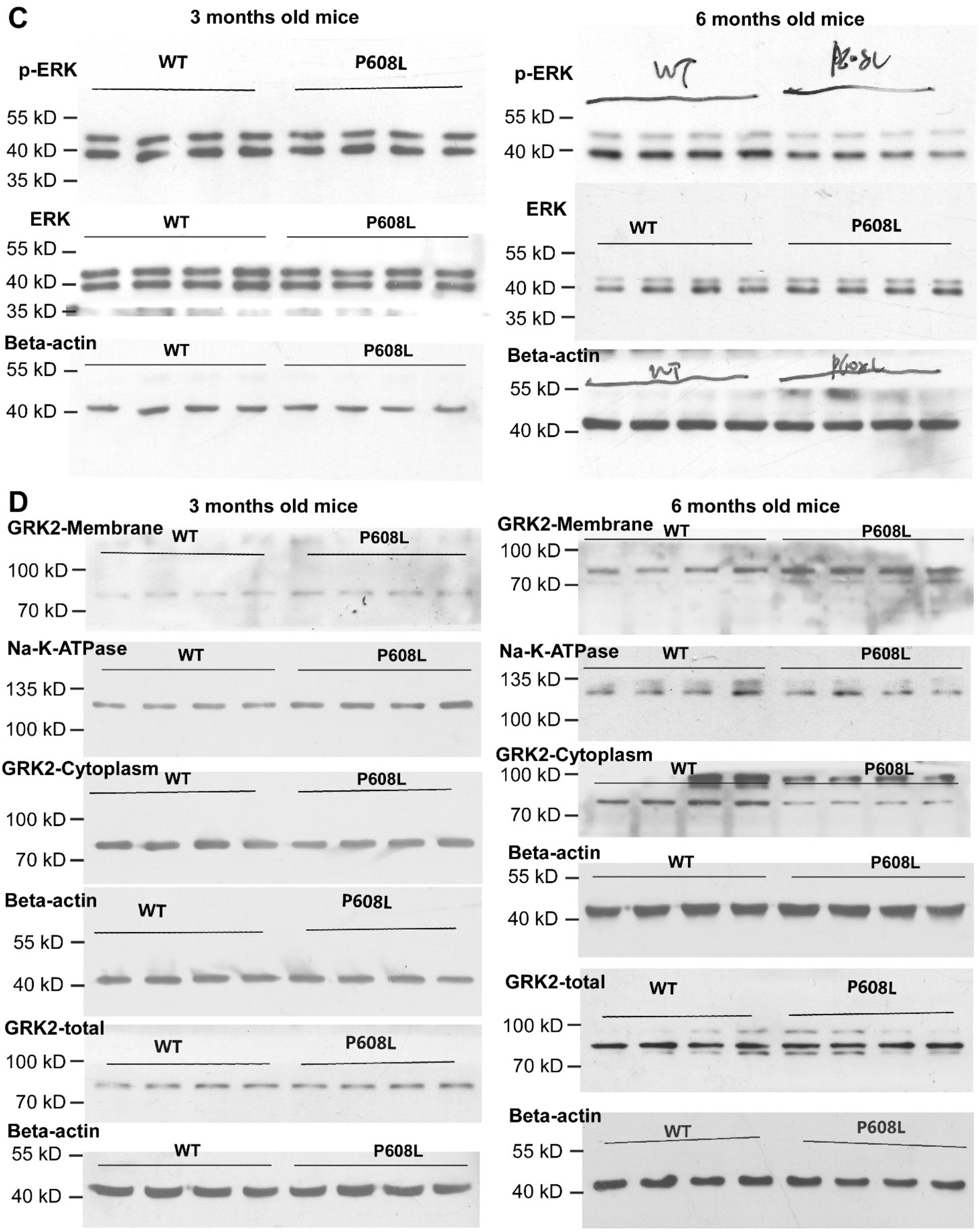
C
3 months old mice
6 months old mice
p-ERK
WT
P608L
55 kD
40 kD
35 kD
ERK
Beta-actin
D
GRK2-Membrane
100 kD
70 kD
Na-K-ATPase
135 kD
GRK2-Cytoplasm
GRK2-total

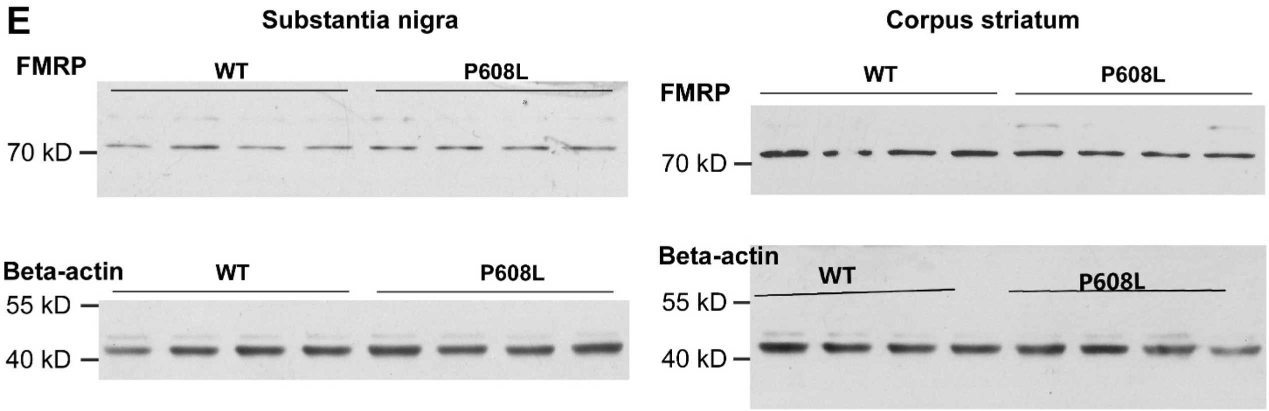

Supplementary Figure 4 Original Western blots in this study. (**A**) Original blots refer to Fig. 4A. (**B**) Original blots refer to Fig. 4D. (**C**) Original blots refer to Fig. 5D. (**D**) Original blots refer to Fig. 6B. (**E**) Original blots refer to supplementary Fig. 3B.
